# Beliefs in Misinformation About COVID-19 and the Russian Invasion of Ukraine Are Linked: Evidence From a Nationally Representative Survey Study

**DOI:** 10.2196/62913

**Published:** 2025-03-10

**Authors:** Dominika Grygarová, Marek Havlík, Petr Adámek, Jiří Horáček, Veronika Juríčková, Jaroslav Hlinka, Ladislav Kesner

**Affiliations:** 1 Center for Advanced Studies of Brain and Consciousness National Institute of Mental Health Klecany Czech Republic; 2 Third Faculty of Medicine Charles University Prague Czech Republic; 3 First Faculty of Medicine Charles University Prague Czech Republic; 4 Institute of Computer Science Czech Academy of Sciences Prague Czech Republic; 5 Faculty of Arts Masaryk University in Brno Brno Czech Republic

**Keywords:** misinformation, COVID-19, war in Ukraine, political trust, digital media, belief rigidity, vaccine hesitancy, war, political, trust, belief, survey, questionnaire, national, false, association, correlation, correlation analysis, public opinion, media, news, health information, public health, COVID, misinformation, propaganda

## Abstract

**Background:**

Detrimental effects of misinformation were observed during the COVID-19 pandemic. Presently, amid Russia’s military aggression in Ukraine, another wave of misinformation is spreading on the web and impacting our daily lives, with many citizens and politicians embracing Russian propaganda narratives. Despite the lack of an objective connection between these 2 societal issues, anecdotal observations suggest that supporters of misinformation regarding COVID-19 (BM-C) have also adopted misinformation about the war in Ukraine (BM-U) while sharing similar media use patterns and political attitudes.

**Objective:**

The aim of this study was to determine whether there is a link between respondents’ endorsement of the 2 sets of misinformation narratives, and whether some of the selected factors (media use, political trust, vaccine hesitancy, and belief rigidity) are associated with both BM-C and BM-U.

**Methods:**

We conducted a survey on a nationally representative sample of 1623 individuals in the Czech Republic. Spearman correlation analysis was performed to identify the relationship between BM-C and BM-U. In addition, multiple linear regression was used to determine associations between the examined factors and both sets of misinformation.

**Results:**

We discovered that BM-C and BM-U were moderately correlated (Spearman ρ=0.57; *P*<.001). Furthermore, increased trust in Russia and decreased trust in the local government, public media, and Western allies of the Czech Republic predicted both BM-C and BM-U. Media use indicating frustration with and avoidance of public or mainstream media, consumption of alternative information sources, and participation in web-based discussions indicative of epistemic bubbles predicted beliefs in misinformation narratives. COVID-19 vaccine refusal predicted only BM-C but not BM-U. However, vaccine refusers were overrepresented in the BM-U supporters (64/161, 39.8%) and undecided (128/505, 25.3%) individuals. Both beliefs were associated with belief rigidity.

**Conclusions:**

Our study provides empirical evidence that supporters of COVID-19 misinformation were susceptible to ideological misinformation aligning with Russian propaganda. Supporters of both sets of misinformation narratives were primarily linked by their shared trust or distrust in the same geopolitical actors and their distrust in the local government.

## Introduction

During the COVID-19 pandemic, many countries worldwide have experienced an increase and acceleration in the spread of conspiracies, hoaxes, misinformation, and intentionally disseminated disinformation [1,2]. A large body of scientific research has demonstrated the detrimental effects of the infodemic on vaccine hesitancy worldwide [3,4], hateful and divisive rhetoric [5], politicization of the issue [6], and radicalization [7].

Social epistemic structures known as echo chambers, which primarily emerge in web-based communities where members reinforce their shared views while actively discrediting other relevant voices [8], have been frequently identified as primary digital channels reinforcing beliefs in misinformation and fueling radicalization [9,10]. Similarly, in the Czech Republic, misinformation narratives have been monitored in web-based communities [11], as well as in chain emails, that have been massively forwarded [12,13]. The main COVID-19 misinformation narratives encompassed a wide range of claims, including the pandemic being a hoax, the assertion that the virus is not dangerous or was artificially developed, and the belief that vaccines are harmful, while PCR tests, face masks, and other preventive measures against COVID-19 pandemic are ineffective [14].

Apart from the spread of misinformation—false information disseminated without the intent to deceive—fueled by the uncertainty of pandemic developments and negative emotions on social media [15], it has been suggested that the issue of COVID-19 pandemic has also been “hijacked” and used by disinformation campaigns conducted for monetary [16] or political purposes [17]. Previous studies have indicated that worries about the harmful effects of vaccination and distrust in Western pharmaceutical companies and politicians have been exploited and reinforced by Russian disinformation campaigns, aiming to undermine public support for state authorities [18]. The Czech Security Information Service reported that pro-Russian activists, promoting antivaccination attitudes and pro-Russian narratives, used COVID-19 pandemic as a useful topic for spreading conspiracies and disinformation [13]. These activists operated largely in symbiosis with the anti–COVID-19 measures movement, particularly on Czech language fringe news websites [13] labeled “disinformation” or “antisystem” websites by media experts [12].

Another massive wave of infodemic began to spread after the Russian invasion of Ukraine in February 2022 [19]. The war has become a new global threat, dominating media coverage and social media attention. Consequently, the focus on COVID-19 pandemic has receded, along with COVID-19 misinformation in the web-based environment [20]. In the Czech Republic, misinformation, including pro-Russian narratives about the conflict in Ukraine and hostile targeting Ukrainian refugees, has spread on “antisystem” websites [21]. These narratives also proliferated via chain emails, which have steeply increased in number after the invasion [20], in social media communities [22], as well as in web-based discussions under web news articles, where increased troll and bot activity has been observed [20,21]. A direct comparison of fact-checking publications revealed that while hoaxes related to both COVID-19 pandemic and the Ukraine war were predominantly disseminated via social media, they differed in their preferred format. Fabricated content was more common in pandemic-related hoaxes, whereas out-of-context images were prevalent in disinformation surrounding the Russia-Ukraine war [23]. The flood of web-based disinformation during both COVID-19 pandemic and the Russian invasion of Ukraine galvanized fact-checking and verification efforts [24-26].

While previous research has shown that individuals who believed in COVID-19 conspiracy theories were more prone to believe in other unrelated, broader conspiracies [27-29], it remains an open question whether those who believe in misinformation about COVID-19 pandemic are also more susceptible to believe politically ideological misinformation. This question has become pressing since the onset of the Russian invasion of Ukraine and the massive spread of disinformation aligned with Russian propaganda. Such disinformation mixes elements of strategic narratives rooted in historical revisionism, imperial mythology, and war memories with factual lies and misinterpretations, aiming to manipulate public opinion and influence political decisions in European Union (EU) and North Atlantic Treaty Organization (NATO) member states [30]. Comparisons have been drawn between the disinformation narratives related to COVID-19 pandemic and those related to the Russia-Ukraine war [14,23]. Anecdotal observations suggest that individuals sharing rigid beliefs in misinformation narratives about COVID-19 pandemic (BM-C) may have also adopted beliefs in misinformation about the Russian invasion of Ukraine (BM-U), and that they tend to use specific digital media channels while avoiding public and mainstream media and share antisystem attitudes and political orientation toward Russia [21]. However, no empirical research has examined this social phenomenon population-wide. Therefore, to validate or refute these observations, we conducted a nationwide representative cross-sectional survey of the Czech Republic.

The first aim of this study was to determine whether there is an association between respondents’ endorsement of the 2 sets of misinformation narratives (BM-C and BM-U).

- Hypothesis 1: There is a correlation between BM-C and BM-U.

The second aim was to examine associations between beliefs in the 2 sets of misinformation (BM-C and BM-U) and factors anecdotally observed or suggested in both contexts. Media monitoring and official reports have indicated that both sets of misinformation have been spreading through specific digital media channels, such as web-based discussions and web-based bubbles or echo chambers, political chain emails, and antisystem websites with political leanings toward Russia [13,21]. However, it remains unknown whether users of these channels are significantly more likely to believe the misinformation and to trust specific geopolitical powers on a nationwide scale. Therefore, we examined associations between (2a) political trust and the 2 sets of misinformation, as well as associations between (2b) media use factors and the 2 sets of misinformation.

- Hypothesis 2a: Distrust in the Czech government’s decisions and public media, trust in Russia, and distrust in Russia’s geopolitical opponents and Western allies of the Czech Republic (US, EU, and NATO) are shared factors that explain both BM-C and BM-U.
- Hypothesis 2b: The use of antisystem websites, emails, and social media as information sources, along with participation in web-based discussions and engagement in web-based bubbles, explains BM-C and BM-U.

The third aim of this study was to examine whether BM-C and BM-U are connected to COVID-19 vaccine refusal. Determining that this factor explains not only BM-C but also BM-U would indicate that this specific health-related behavior significantly reflects the politicization of the COVID-19 issue to such an extent that it increased susceptibility to ideological misinformation.

- Hypothesis 3: COVID-19 vaccine refusal explains both BM-C and BM-U.

In addition, we aimed to test whether beliefs in the 2 categories of misinformation are associated with belief rigidity. The underlying assumption is that individuals who endorse misinformation place greater emphasis on the importance of these beliefs, as they often provide complex collective narratives and transcend mere opinions on specific health, societal, or political issues. Rather, they may become a belief system infused with moral convictions, which tends to be fixed and rigid [31,32]. Belief rigidity has been connected to echo chambers [8,33], conspiracy thinking [34], and polarization [31,35,36].

- Hypothesis 4: Belief rigidity explains both BM-C and BM-U.

## Methods

### Procedure

The data were collected from April 25 to May 5, 2022, at the time when COVID-19 pandemic had subsided and 2 months after the start of the Russian invasion of Ukraine. The cross-sectional survey was completed by members of the Czech National Panel [37] as a part of a longitudinal study [38], using the standardized computer-assisted web interviewing method. Participation was voluntary, with financial compensation. The mean completion time of the survey was approximately 11 minutes, and participants were informed in advance about the length. The survey included sociodemographic data (gender, age, level of education, region of residence, and household income), as well as questions about beliefs in misinformation regarding COVID-19 pandemic and the Russian invasion of Ukraine, media use, political trust, belief rigidity, and whether and how many times they have been vaccinated against COVID-19. Only self-reported measures were used. To ensure the protection of personal information, all collected data were securely stored in an encrypted, password-protected institutional database hosted on National Institute of Mental Health servers. Only authorized personnel had access to the data. Any personal identifiers were anonymized during data processing to prevent unauthorized access or identification of participants.

### Participants

Participants of the longitudinal study [38] were invited to participate in this study. We received responses from 1623 respondents (return rate: 55% of 2950 invited; 839/1623, 51.7% women) aged between 20 and 91 years (mean 55.04, SD 15.55). The proportions of participants’ attained educational levels were as follows: 4.6% (76/1623) elementary school education, 29.1% (472/1623) certificate of apprenticeship, 36.2% (587/1623) high school education, and 30.2% (490/1623) university degree. The sample was constructed to be quota-representative of the adult population of the Czech Republic. To ensure repeated participation of various sociodemographic groups, it was necessary to adjust the current sample through poststratification weighting. This adjustment was based on current population distributions (using data from the Czech Statistical Office) for the following characteristics: gender, age, education, size of place of residence, region, crosscutting of age and education, crosscutting of age and gender, and employment status. The inclusion criteria were knowledge of the Czech language and being older than 18 years.

### Measures

#### Beliefs in Misinformation Narratives

To measure BM-C and BM-U, we developed 2 questionnaires. The questionnaires were constructed based on the main misinformation related to COVID-19 published by the Center Against Hybrid Threats within the Ministry of the Interior of the Czech Republic [39]. The Ministry reported that such narratives had been spread in an attempt to exploit societal issues in accordance with the interests of foreign powers. We reduced the number of items from the original 15 to 6 based on results from our pilot study (N=423), excluding items according to item analysis, exploratory factor analysis (EFA), and the results of the Cronbach α coefficient. BM-C items are shown in Textbox 1. Similarly, the BM-U questionnaire was constructed, using the prevalent misinformation narratives related to the Russian invasion in Ukraine at the time of the study [40]. We selected 4 items from the original 8 based on pilot data according to the same procedure as in BM-C. BM-U items are shown in Textbox 1. Both questionnaires showed good internal consistency in both the pilot study (BM-C: Cronbach α=0.953; BM-U: Cronbach α=0.932) and in this study (BM-C: Cronbach α=0.846; BM-U: Cronbach α=0.891). Participants rated the items on a 5-point scale (1: “I do not agree at all”—5: “I completely agree”).

**Items for beliefs in misinformation narratives (beliefs in misinformation narratives about COVID-19 pandemic [BM-C] and beliefs in misinformation about the Russian invasion of Ukraine [BM-U]).**

Evaluate the extent to which you agree or disagree with the following statements.

BM-C

- "Western pharmacological vaccine companies are untrustworthy."
- "Vaccines are dangerous for the vaccinated."
- "The discrimination against Russian and Chinese vaccines is largely driven by political reasons."
- "The coronavirus was developed artificially, perhaps as a biological weapon."
- "The epidemic is fake, the situation has never been so serious."
- "Epidemic measures were ineffective and were counterproductive."

BM-U

- "The demilitarisation and de-Nazification of Ukraine is a legitimate objective for the Russian military operation in Ukraine."
- "The civilian casualties on the Ukrainian side are deliberately exaggerated by the European media."
- "Ukraine is developing banned biological weapons on its territory."
- "NATO and Western countries are exploiting Ukraine to serve their own interests."

#### COVID-19 Vaccination

Participants were asked whether and how many times they had been vaccinated against COVID-19 (0, 1, 2, or 3 times). It should be noted that at the time of the survey, the Ministry of Health of the Czech Republic recommended 3 doses of the vaccine.

#### Media Use

We used an adapted version of the media use questionnaire [41]. We omitted some items and included additional ones, while also rewording some items to better suit the research objectives of measuring media behavior and media effects that may be indicative of or contribute to the spread of misinformation. To compare responses to the 2 societal issues, we used identical wording for questions related to the COVID-19 pandemic (C), and the Russian invasion of Ukraine (U), with only a difference in the topic and time frame being questioned (eg, “How often did you search for news regarding COVID-19 at the height of the pandemic?” or “How often did you search for news on the Russian invasion of Ukraine last month?”). The mirrored items were placed in different locations within the questionnaire and never in sequence. The newly developed measures were tested in a pilot survey conducted via Facebook in April 2022 (N=423; response rate: 51.8% of 817 invited). Respondents were asked about their frequency of use of media channels categorized as public media, mainstream news websites [42], and those that have been previously connected to spreading misinformation: emails as a source of information (possibly indicating political chain emails), YouTube, social media, and “anti-system websites” that have been identified as such by various media experts [12,42]. However, at the time of our survey, in reaction to the Russian invasion of Ukraine and the uncertain development of the situation, most of the antisystem websites were evaluated as a threat to national security and were officially banned in the Czech Republic due to their open promotion of Russian disinformation narratives. Only 1 functioning, moderate news website, remained in our survey. Participants were also asked about their engagement in web-based discussions and web-based bubbles related to C/U. Furthermore, we decided to examine several other aspects of media use—searching and sharing the news (C/U), respondents’ interest in the 2 topics (C/U), and their frustration with public and mainstream media.

#### Political Trust

Perceptions of trust in the (1) Czech government and (2) public media were assessed in relation to both issues (C/U). Due to the high correlation of items 1 (C) and 2 (C) (*r*=0.783, n=1623; *P*<.001), as well as items 1 (U) and 2 (U) (*r*=0.849, n=1623; *P*<.001), we summed the items in 1 score for each topic: trust in the Czech government and public media regarding COVID-19 (*Trust in CZ-C*); trust in the Czech government and public media regarding Russian invasion of Ukraine (*Trust in CZ-U*)*.* In addition, distrust in foreign geopolitical actors (Russia, United States, China, EU, and NATO) and belief rigidity was assessed. Detailed descriptions of the survey items and response scales for media use, political trust, and belief rigidity are shown in Multimedia Appendix 1.

### Statistical Analysis

All data were analyzed using R software (R Core Team). The significance level was set at *P*≤.05. Poststratification weighting was applied using a quadratic programming algorithm based on current population distributions of the following characteristics: gender, age, education, region, residence size, job status, interaction between age and education, and interaction between age and gender. Descriptive statistics were used for demographic description. Shapiro-Wilk test did not confirm the normal distribution of BM-C and BM-U. EFA was conducted on both BM-C and BM-U items to uncover the latent structure based on interdependence between the items. The primary aim of the EFA was to clearly differentiate COVID-related and ideological items, ensuring that the correlation between BM-C and BM-U scales is not influenced by the ideological items possibly present in BM-C.

As the data were nonparametric, we used Spearman correlation to determine the relationship between BM-C and BM-U (Hypothesis 1). Multiple linear regression models were used to reveal the relationships between the examined factors according to Hypotheses 2-4 (COVID-19 vaccine refusal, media use, political trust, and belief rigidity) and beliefs in BM-C and BM-U. For the multiple linear regression models, we used normalization of nonparametric right-skewed data by square root. Two distinct models were constructed, 1 for BM-C and 1 for BM-U (dependent variables), with COVID-19 vaccine refusal, media use, political trust, and belief rigidity as independent variables. We also controlled for demographic characteristics (age, gender, education, and income). To compare the predictive power of the independent variables, we used a feature scaling approach. Specifically, we used normalization to standardize all continuous input variables to a uniform range of 1-5. This step guarantees comparability and stability in the regression analysis, establishing a standardized input space for the model and enabling the evaluation of the effect of each variable. However, categorical variables were maintained in their original scale to preserve their interpretability and intrinsic categorical distinctions.

### Ethical Considerations

The procedure performed in this study was in accordance with the ethical standards of the institutional research committee and with the 1964 Helsinki Declaration and its later amendments. The study was approved by the ethics committee of the National Institute of Mental Health, Czech Republic (reference no. 181/21). The data were anonymized. Respondents were compensated by the Czech National Panel at a standard rate of 1 CZK (US $0.041) per minute for completing the questionnaire. The compensation was provided as credit, which could be transferred to a bank account, redeemed for a material reward, or donated to charity. In addition, 2 randomly selected participants had the chance to win a tablet. All participants provided informed consent. They were informed about the purpose of the study. Furthermore, they were informed that the data would be accessible only to authorized research staff and the principal investigator, whose name and contact information were provided for any follow-up questions or concerns. Participants were assured that their participation was voluntary.

## Results

### Exploratory Factor Analysis

EFA was conducted using parallel analysis to identify the underlying structure of the BM-C and BM-U items. Two factors were extracted, explaining 58.7% of the total variance, with factor 1 accounting for 32.4% of the variance and factor 2 accounting for 26.3%. The overall Kaiser-Meyer-Olkin measure of sampling adequacy was 0.91, indicating that the data were highly suitable for factor analysis. The Bartlett sphericity test (*χ*^2^_45_=9338; *P*<.001) further confirmed the appropriateness of conducting EFA. An Oblimin rotation was applied to enhance interpretability, allowing for correlations between factors. The first factor, labeled “Ideological,” included all BM-U items and BM-C item 3 (“The discrimination against Russian and Chinese vaccines is largely driven by political reasons”). The second factor, labeled “COVID,” comprised all remaining BM-C items (except item 3). Due to its significant loading on the ideological factor and theoretical considerations, BM-C item 3 was excluded from further analysis. Factor loadings are shown in Multimedia Appendix 2.

### Correlation Between BM-C and BM-U and Descriptive Statistics for BM-C and BM-U

**Figure 1:**
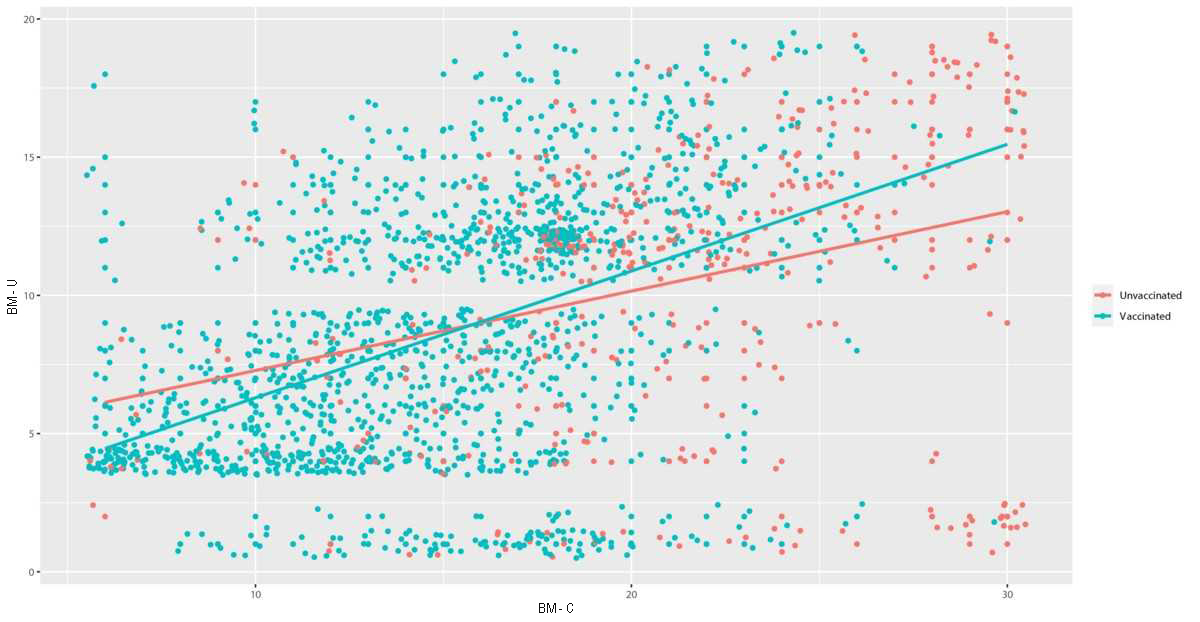
Distribution of unvaccinated (shown in red) and vaccinated (shown in cyan) against COVID-19 pandemic in relation to beliefs in misinformation regarding COVID-19 (BM-C) and the war in Ukraine (BM-U). The x-axis represents BM-C total score, and the y-axis represents BM-U total score.

A moderate positive correlation was found between BM-C and BM-U (Spearman ρ=0.57; *P*<.001). For a more straightforward description of BM-C and BM-U, we considered 4 points (“I rather agree”) and 5 points (“I completely agree”) as an indication of belief in misinformation (*supporters*). Those who rated 3 points (“I neither agree nor disagree”) were considered undecided whether they believe in misinformation or not (*undecided*). Those who rated 1 (“I completely disagree”) or 2 (“I rather disagree”) were considered *opponents* who do not endorse misinformation narratives. According to this grouping based on cumulative scores, the prevalence of BM-C supporters was 13.4% (217/1623), and the prevalence of BM-U supporters was 9.9% (161/1623). There were 50% (812/1623) of undecided respondents for BM-C and 31.1% (505/1623) for BM-U. The demographic description showed that supporters in BM-C were most represented in apprenticeship education degree (88/217, 41%), followed by high school degree (77/217, 36%) and university education level (42/217, 19%), with lowest numbers in elementary education level (10/217, 5%). BM-C opponents were most prevalent in the university education level (243/594, 40.9%). Supporters of BM-U were most prevalent in apprenticeship education level (60/505, 37%), followed by high school degree (54/161, 34%) and university degree (39/141, 24%). BM-U opponents were most prevalent in high school (357/957, 37.3%) and university education (342/957, 35.7%), followed by apprenticeship education (223/957, 23.3%). Overall, supporters and undecided both for BM-C and BM-C were less prevalent in the university education level and more in the apprenticeship education level compared with nonsupporters. Regarding household income, supporters and undecided (both for BM-C and BM-C) were represented less in the high-income group and more in the below poverty line income group compared with opponents. In terms of gender, noticeable differences were found in the undecided groups, particularly in BM-U, with female participants representing a higher proportion (304/505, 60.2%). Conversely, male participants were more prevalent among BM-U supporters (98/161, 61%). Differences in age compared with an average of the whole sample (mean 55.04, SD 15.56) were observed only in BM-U supporters, who were older (60.9 years), and BM-U opponents, who were younger (49.3 years). Vaccine refusers were minimally represented in BM-C opponents (33/594, 6%), more in BM-C undecided (146/812, 18%), and most in BM-C supporters (142/217, 65%). Moreover, 34.6% (75/217) of BM-C supporters were vaccinated despite their beliefs. Regarding BM-U, vaccine refusers were most represented in BM-U supporters (64/161, 40%), followed by BM-U undecided (128/505, 25.3%), with the lowest numbers in BM-U opponents (129/957, 13.5%; Figure 1). The descriptive statistics are shown in Table 1.

**Table 1:** Sociodemographic characteristics of BM-C^a^ and BM-U^b^.

Sociodemographic variables	Opponents BM-C	Undecided BM-C	Supporters BM-C	Opponents BM-U	Undecided BM-U	Supporters BM-U
Male + female, n (%)	594 (36.6)	812 (50)	217 (13.4)	957 (59)	505 (31.1)	161 (9.9)
Female, n (%)	267 (44.9)	459 (56.5)	113 (52.1)	472 (49.3)	304 (60.2)	63 (39.1)
Male, n (%)	327 (55.1)	353 (43.5)	104 (47.9)	485 (50.7)	201 (39.8)	98 (60.9)
Age (years), mean (SD)	55.02 (16.62)	55.25 (15.11)	54.31 (14.16)	53.25 (15.19)	56.58 (14.89)	60.80 (13.28)
Elementary education, n (%)	17 (2.9)	47 (5.8)	10 (4.6)	35 (3.7)	31 (6.1)	8 (5)
Apprenticeship education, n (%)	119 (20)	265 (32.6)	88 (40.6)	223 (23.3)	189 (37.4)	60 (37.3)
High school education, n (%)	215 (36.2)	295 (36.3)	77 (35.5)	357 (37.3)	176 (34.9)	54 (33.5)
University education, n (%)	243 (40.9)	205 (25.2)	42 (19.4)	342 (35.7)	109 (21.6)	39 (24.2)
Income 1^c^, n (%)	32 (5.4)	57 (7)	26 (12)	55 (5.7)	41 (8.1)	19 (11.8)
Income 2^d^, n (%)	164 (27.6)	268 (33)	81 (37.3)	289 (30.2)	175 (34.7)	49 (30.4)
Income 3^e^, n (%)	231 (38.9)	325 (40)	88 (40.6)	369 (38.6)	211 (41.8)	64 (39.8)
Income 4^f^, n (%)	167 (28.1)	162 (20)	22 (10.1)	244 (25.5)	78 (15.4)	29 (18)
Vaccinated, n (%)	561 (94.4)	666 (82)	75 (34.6)	828 (86.5)	377 (74.7)	97 (60.2)
Unvaccinated, n (%)	33 (5.6)	146 (18)	142 (65.4)	129 (13.5)	128 (25.3)	64 (39.8)

^a^BM-C: beliefs in misinformation narratives about COVID-19 pandemic.

^b^BM-U: beliefs in misinformation about the Russian invasion of Ukraine.

^c^Below poverty line income (below 60% of the median).

^d^Low income (below the median).

^e^Upper middle income (up to 1.5 times the median).

^f^High income (above 1.5 times the median).

### Factors Explaining BM-C

The multiple linear regression model explained 44.92% of the individual differences in BM-C (*F*_30,1592_=45.1; adjusted *R*^2^=0.45; *P*<.001). Descriptions of variables used in the BM-C model are shown in Multimedia Appendix 3. The results showed significant relationships between the 12 examined factors as the independent variables and BM-C total score as the dependent variable (Table 2). Trust in the Czech government and public media, vaccination against COVID-19 pandemic, distrust in Russia, searching for news on COVID-19 pandemic, and participation in web-based discussions predicted lower levels of BM-C. Distrust in the United States, distrust in the EU, frustration from public and mainstream news, rigid beliefs, use of emails as a source of information, sharing COVID-19 news, and engagement in web-based bubbles predicted higher levels of BM-C. Regarding demographic factors, upper middle income (compared with high income), as well as elementary, apprenticeship, and high school education levels (compared with university education level) were associated with increased BM-C. The below poverty line income group (compared with high income) predicted lower levels of BM-C.

**Table 2:** The results of multiple linear regression models for BM-C^a^ and BM-U^b,c^.

Explaining variable	BM-C, coefficient (SE)	BM-C, *t* test (*df*)	BM-C, *P* value	BM-U, coefficient (SE)	BM-U, *t* test (*df*)	BM-U, *P* value
Intercept	2.11 (0.25)	8.48 (1592)	*<.001*	2.30 (0.28)	8.24 (1592)	*<.001*
COVID-19 vaccination	–0.17 (0.01)	–12.93 (1592)	*<.001*	–0.02 (0.01)	–1.55 (1592)	.12
Information from emails	0.04 (0.02)	2.05 (1592)	*.04*	0.04 (0.02)	2.13 (1592)	*.03*
YouTube	0.03 (0.02)	1.79 (1592)	*.07*	–0.02 (0.02)	–1.26 (1592)	.21
Antisystem websites	0.03 (0.02)	1.50 (1592)	.13	0.06 (0.02)	2.92 (1592)	*.004*
Public media	0.03 (0.02)	1.84 (1592)	.07	–0.02 (0.01)	–1.37 (1592)	.17
Mainstream websites	0.02 (0.02)	0.97 (1592)	.33	–0.04 (0.02)	–2.52 (1592)	*.01*
Exposure to social media	–0.03 (0.06)	–0.56 (1592)	.58	–0.07 (0.05)	–1.42 (1592)	.16
Social media information source	–0.001 (0.02)	–0.06 (1592)	.95	0.03 (0.02)	2.01 (1592)	*.045*
Discussions under news	0.02 (0.02)	1.06 (1592)	.29	0.05 (0.02)	2.68 (XX)	*.007*
Discussions on social media	0.32 (0.10)	3.18 (1592)	*.002*	–0.02 (0.15)	–0.12 (1592)	.90
Web-based bubbles	0.17 (0.06)	3.10 (1592)	*.002*	0.03 (0.07)	0.42 (1592)	.68
Search for news	–0.1 (0.03)	–3.81 (1592)	*<.001*	–0.06 (0.02)	–2.30 (1592)	*.02*
Sharing news	0.07 (0.03)	2.47 (1592)	*.01*	0.01 (0.03)	0.48 (1592)	.63
Interest in news	0.01 (0.02)	0.76 (1592)	.45	–0.03 (0.02)	–1.59 (1592)	.11
Frustration from media	0.12 (0.02)	6.27 (1592)	*<.001*	0.10 (0.02)	5.42 (1592)	*<.001*
Trust in Czech government	–0.22 (0.02)	–10.34 (1592)	*<.001*	–0.23 (0.02)	–11.81 (1592)	*<.001*
Distrust in Russia	–0.07 (0.02)	–2.94 (1592)	*.003*	–0.24 (0.02)	–10.26 (1592)	*<.001*
Distrust in United States	0.08 (0.03)	2.33 (1592)	*.02*	0.20 (0.03)	6.63 (1592)	*<.001*
Distrust in EU^d^	0.12 (0.04)	2.86 (1592)	*.004*	0.10 (0.04)	2.65 (1592)	*.008*
Distrust in China	0.02 (0.03)	0.82 (1592)	.41	–0.05 (0.02)	–0.22 (1592)	.82
Distrust in NATO^e^	–0.05 (0.04)	–1.20 (1592)	.23	0.07 (0.04)	1.81 (1592)	.07
Rigid beliefs	0.09 (0.02)	5.07 (1592)	*<.001*	0.08 (0.02)	4.99 (1592)	*<.001*
Income 1 (below poverty line)^f^	–0.18 (0.08)	–2.35 (1592)	*.02*	0.14 (0.07)	2.00 (1592)	*.046*
Income 2 (low)^f^	0.09(0.05)	1.75 (1592)	.08	0.07 (0.05)	1.42 (1592)	.16
Income 3 (upper middle)^f^	0.10 (0.05)	2.04 (1592)	*.04*	0.07 (0.05)	1.48 (1592)	.14
Elementary education^g^	0.31 (0.07)	4.37 (1592)	*<.001*	0.20 (0.07)	3.00 (1592)	*.003*
Apprenticeship education^g^	0.22 (0.05)	3.97 (1592)	*<.001*	0.06 (0.05)	1.14 (1592)	.25
High school education^g^	0.17 (0.05)	3.40 (1592)	*<.001*	0.02 (0.05)	0.32 (1592)	.75
Gender (female)^h^	–0.03 (0.04)	–0.89 (1592)	.37	–0.03 (0.04)	–0.81 (1592)	.42
Age (year)	0.001 (0.001)	1.01 (1592)	.31	0.07 (0.02)	3.23 (1592)	*<.001*

^a^BM-C: beliefs in misinformation narratives about COVID-19 pandemic.

^b^BM-U: beliefs in misinformation about the Russian invasion of Ukraine.

^c^Significant values are italicized.

^d^EU: European Union.

^e^NATO: North Atlantic Treaty Organization.

^f^Contrasted to high-income group.

^g^Contrasted to university degree.

^h^Contrasted to male.

### Factors Explaining BM-U

The multiple regression model explained 62.21% of the variance in BM-U (*F*_30,1591_=90.01; adjusted *R*^2^=0.62; *P*<.001). Descriptions of variables used in the BM-U model are shown in Multimedia Appendix 3. We found significant relationships between the 12 examined factors as independent variables and BM-U total score as the dependent variable (Table 2). Trust in the Czech government and public media, distrust in Russia, consumption of mainstream news websites, and searching for news about the war in Ukraine predicted lower levels of BM-U. Conversely, distrust in the United States, distrust in the EU, frustration from public and mainstream news, consumption of “antisystem websites,” use of emails as a source of information, use of social media as an information source, reading discussions under web news articles, and belief rigidity predicted higher levels of BM-U. Regarding demographic factors, below poverty line income (compared with high income), elementary education level (compared with university education level), and older age were associated with higher levels of BM-U.

## Discussion

### Principal Findings

Our study provides evidence of a connection between beliefs in COVID-19 misinformation (BM-C) and misinformation regarding the Russian invasion of Ukraine (BM-U) by identifying a correlation between these 2 sets of beliefs and several shared factors. Regarding political trust, higher trust in Russia and lower trust in local government, public media, and Western allies of the Czech Republic (the EU and the United States) were revealed as strong predictors of both BM-C and BM-U. In addition, frustration with public and mainstream media, using emails as a source of information––possibly indicating chain emails––and reduced frequency in searching for news related to COVID-19 pandemic or war in Ukraine, predicted both BM-C and BM-U. We also identified media use patterns commonly associated with the spread of misinformation, which predicted either BM-C or BM-U. These included participation in web-based bubbles, engagement in discussions under web news articles, use of antisystem websites, avoidance of mainstream media, use of social media as an information source, and sharing news. In addition, belief rigidity was a significant predictor for both BM-C and BM-U.

### Correlation Between BM-C and BM-U

A moderate positive correlation discovered between BM-C and BM-U supports our hypothesis, indicating that a significant number of individuals believing in COVID-19 misinformation have also adopted ideological misinformation regarding the Russian invasion of Ukraine. This extends previous findings that beliefs in COVID-19 conspiracies correlate with beliefs in other, broader and unrelated conspiracies [27,28] to the politicized side of COVID-19 misinformation, which increased susceptibility to ideological misinformation aligning with Russian propaganda. Our finding provides further evidence for the so-called “conspiracy singularity” [43] suggesting the tendency of actors to spread and interconnect various conspiracy theories [44,45]. For instance, the same actors who spread COVID-19 conspiracies before the Russian invasion of Ukraine later disseminated anti-NATO and pro-Russian narratives in Finland [46] and Slovakia [47]. Our findings thus corroborate similar phenomena observed beyond the context of the Czech Republic and may provide further insights into the mechanisms by identifying underlying factors revealed in our analyses, which are discussed in the sections “Associations of Political Trust and Beliefs in Misinformation,” “Associations of Media Use and Beliefs in Misinformation Narratives,” “COVID-19 Vaccine Refusal,” and “Belief Rigidity.”

### Associations of Political Trust and Beliefs in Misinformation

Our finding that lowered trust in governmental decisions and public media was associated with both increased BM-C and BM-U supported our hypothesis. Moreover, it was the strongest predictor explaining both BM-C and BM-U. It is in line with previous research linking distrust in public institutions to COVID-19 misinformation beliefs [48-51]. While most previous findings on associations between beliefs in COVID-19 misinformation and political attitudes report that conservatism is associated with increased susceptibility to misinformation [52-54], we did not inquire about partisanship but rather about trust in geopolitical powers. Our results showing increased trust in Russia in higher levels of both BM-C and BM-U indicate a leaning toward this geopolitical power in supporters of both sets of misinformation. In addition, we observed increased distrust toward the Czech Republic’s geopolitical allies and Russia’s main opponents—the United States and the EU—among individuals with higher levels of both BM-C and BM-U. While this ideological inclination is not surprising regarding BM-U, which openly promotes Russian propaganda, it is not as readily apparent in the case of BM-C. However, our result aligns with previous research that has suggested the role of Russian disinformation campaigns in supporting the antivaccination movement [18,55,56].

Our findings can thus be contextualized in light of the goals of Russia’s hybrid war strategy, which aims to continually undermine the trustworthiness and legitimacy of foreign governments in the eyes of the target population by warping their beliefs, thoughts, decisions, and behavior over the long term [57]. The goal of this tactic is to gradually reconstruct the target population’s prior beliefs in favor of Russia [58,59]. However, our study cannot establish a causal relationship in terms of direct influence of Russia’s disinformation campaigns. The inclination toward Russia may also have deep historical roots, as the Czech Republic––former Czechoslovakia––was part of the Eastern Bloc under the direct influence of the Soviet Union for 4 decades. Increased trust in Russia may also represent an alternative to the current Western orientation of the Czech Republic as a member of the EU and NATO, reflecting a broader, socially driven epistemic mistrust that manifests in the rejection of authoritative information, as suggested by the socioepistemic model of belief in conspiracy theories [60].

### Associations of Media Use and Beliefs in Misinformation Narratives

All of the identified media use factors linked to either BM-C or BM-U provided support for our hypothesis regarding media use, formulated based on previous observations and theoretical or empirical associations with the dissemination of misinformation. However, it is noteworthy that not all of the examined factors demonstrated significant relationships with both BM-C and BM-U. The strongest media factor associated with higher levels of both beliefs was identified as frustration with the public and mainstream media. While previous research has established this factor as a predictor of higher anxiety and depression levels during the COVID-19 pandemic [41], our study extends its relevance to the context of misinformation susceptibility. This observation is complemented by another finding, which links less frequent searches for COVID-19 news with higher BM-C levels, and less frequent consumption of mainstream media and searches for the news about the war in Ukraine with BM-U. These findings align with previous research [1,61,62] and suggest that supporters of misinformation narratives engage in avoidance behavior, possibly due to their mistrust in information they perceive as misrepresented in public and mainstream media.

On the other hand, supporters of BM-C and BM-U showed higher engagement with other media channels. Specifically, there was an association between obtaining news information from emails––possibly indicating chain emails––and both BM-C and BM-U. In addition, reading discussions under web news articles and consuming information from antisystem websites was positively associated with BM-U. These findings corroborate observations regarding the role of such media channels in disseminating misinformation content and the susceptibility of their consumers to misinformation [13,20].

Next, the positive relationship between obtaining information from social media and increased BM-U, as well as the association between engagement in web-based bubbles and increased BM-C, indicates that the social media environment contributed to the spread of misinformation and their users’ endorsement, as suggested by previous research [1,51,63-66]. While we acknowledge the limitations of the web-based survey method in assessing the phenomenon of web-based (epistemic) bubbles or echo chambers, it is plausible to assume that this phenomenon may have indeed been reflected in our results, as it aligns with prior findings [8-10,64].

Conversely, the negative relationship of engagement in discussions on social media and BM-C, as well as the lack of discernable associations between cumulative exposure to social media and BM-C/BM-U, underscores the reductive conclusions of associating social media platforms solely with the spread of misinformation. Indeed, social media offers users engagement in socializing and discussing a diverse array of content, as well as a broad spectrum of viewpoints on sociopolitical issues. Notably, in the context of nondemocratic regimes, digital media often serves as a primary source of obtaining reliable information. Research in nondemocratic regimes indicates that the use of digital media correlates with diminished adherence to misinformation, contrasting with users reliant solely on official information channels [67].

Our next finding of a positive association between sharing news and heightened levels of BM-C indicates that BM-C supporters demonstrated a propensity for active engagement with digital media. Speculatively, this could be due to heightened arousal triggered by specific content, frustration, or a sense of moral obligation to disseminate the alternative information on social media, perceived as accurate, compared with information reported by public and mainstream media, perceived as misleading or incomplete [68]. This inference is drawn from previous research indicating that the perceived accuracy of content significantly influences the likelihood of its sharing by users [69]. While our study did not directly explore the specific content shared by respondents, it is pertinent to note that previous studies have demonstrated that misinformation tends to be inherently more frequently shared than other types of news [69].

### COVID-19 Vaccine Refusal

Our finding that vaccine refusal was a strong factor associated with BM-C supports our hypothesis and aligns with extensive prior research linking exposure to COVID-19 misinformation to COVID-19 vaccine hesitancy [48,62,70-73]. Our finding provides further evidence that COVID-19 vaccine refusal is a behavioral indicator of diverse attitudes that transcend medical concerns. However, it is important to note that 34.6% of BM-C supporters (75/217) reported being vaccinated, indicating a divergence from their beliefs. They may ultimately yield to social pressure and decide to get vaccinated, considering the practical difficulties posed by remaining unvaccinated in their daily lives during the pandemic.

Contrary to our hypothesis, COVID-19 vaccine refusal was not associated with BM-U, suggesting that this health-related behavior is a broader phenomenon that includes vaccine hesitancy due to health reasons, medical concerns, simple reluctance, and other factors. We conclude that vaccine refusal should not lead to the reductionist conclusion that COVID-19 vaccination was entirely politicized. However, we observed a higher prevalence of vaccine refusers in BM-U supporters (64/161, 39.8%), followed by BM-U undecided (128/505, 25.3%), with the lowest numbers in BM-U opponents (129/957, 13.5%). Special attention should be given to the BM-U undecided group, requiring longitudinal monitoring to assess whether they might become new adherents of BM-U.

### Belief Rigidity

Our additional finding of a positive association between the rigidity of one’s beliefs regarding sociopolitical issues with both BM-C and BM-U indicates that those who adhere to the alternative interpretations of both sociopolitical issues tend to harbor more fixed and rigid opinions than those who do not support such interpretations. Our finding is consistent with previous studies connecting belief rigidity to conspiratorial thinking [34] and beliefs in misinformation propagated through social media [74]. Rigid beliefs have been found to facilitate group cohesion, partisanship, polarization, and extremism [31,35,75]. It is thus plausible that beliefs such as BM-C or BM-U may serve as a group-shared alternative “truth” while being shared through the digital media environment as identified in our analysis. Furthermore, it is in line with our other finding (discussed in the "Associations of Media Use and Beliefs in Misinformation Narratives" section) indicating avoidance of public and mainstream information sources. This pattern is consistent with previous research suggesting that belief rigidity is strengthened when individuals isolate themselves from contradictory information, thus reinforcing their confirmation bias [10].

### Conclusions

Our findings support the hypothesis that individuals who endorsed COVID-19 misinformation were more susceptible to ideological misinformation, aligning with Russian propaganda. Supporters of both misinformation narratives shared common traits, including heightened distrust of local government, public media, the United States, and the EU, along with increased trust toward Russia. They also exhibited increased belief rigidity and demonstrated several common media use patterns, previously linked to the spread of misinformation. To gain a deeper understanding of these phenomena, longitudinal monitoring is essential. By tracking the development of BM-C, BM-U, and the examined factors over time, causal relationships can be uncovered.

### Limitations

The primary shortcoming of this study was the constraint imposed by the short survey format. Due to time limitations, it was not feasible to use longer standardized questionnaires such as the Belief Rigidity Scale. Instead, we opted for a single statement specifically related to societal issues, such as politics, war, and pandemics, and we considered this finding as supplementary. On the other hand, we chose to investigate media use in more detail with practical implications in mind, aiming to identify specific media channels where misinformation is prevalent for targeted recommendations. However, some aspects of the media environment, such as web-based communities with an echo chamber effect and chain emails, were challenging to assess via survey. Consequently, our findings regarding these information sources should be interpreted with caution. In addition, while we acknowledge the availability of standardized COVID-19 conspiracy or misinformation scales, our objective was to study COVID-19 misinformation prevalent in the local context of the Czech Republic as identified by previous analytical sources.

## Data Availability

The dataset generated during this study is available in the OSF data repository (osf.io/wtuqj).

## Appendix

Multimedia Appendix 1 Items and response scales for variables on media use, political trust, and belief rigidity.

Multimedia Appendix 2 Factor loadings of BM-C and BM-U items resulting from exploratory factor analysis.

Multimedia Appendix 3 Descriptions of variables used in multiple linear regression models for BM-C and BM-U.

## Abbreviations

BM-C: beliefs in misinformation narratives about COVID-19 pandemic
BM-U: beliefs in misinformation about the Russian invasion of Ukraine
EFA: exploratory factor analysis
EU: European Union
NATO: North Atlantic Treaty Organization
