# Supplementary material for: Beliefs in Misinformation About COVID-19 and the Russian Invasion of Ukraine Are Linked: Evidence From a Nationally Representative Survey Study
Source: JMIR Infodemiology. 2025 Mar 10;5:e62913. doi: 10.2196/62913 (PMC11956375; doi:10.2196/62913)
Supplement: Multimedia Appendix 1 [file infodemiology_v5i1e62913_app1.docx]

| Variable | Item | Response scale |
| --- | --- | --- |
| **Media use** |  |  |
|  | “How often do you watch/read/ listen to news from the following media outlets?” | 1=”never”, 5=“more than two times a day” |
| YouTube | “YouTube” |  |
| Anti-system websites | “Parlamentní listy” |  |
| Public media | “Czech television/ Czech Radio” |  |
| Mainstream websites | “At least one of the following media outlets: Lidové noviny/ Hospodářské noviny/ Právo, Deník/ MF DNES” |  |
| Social media as info. source | „How often do you obtain information about current societal events from social media?“ | 1=”never”, 5=”very often” |
| Emails | „How often do you obtain information about current societal events from emails?“ | 1=”never”, 5=”very often” |
| Exposure to social media | “Estimate how much time you usually spend on social media each day in hours and minutes.” | “hours and minutes per day” |
| Discussions under web news articles | “How often do you read reader comments under articles on news websites?” | 1=”never”, 5=”very often” |
| Discussions on social media (C) | “Did you participate in online discussions about COVID-19 during the height of the pandemic?” | “yes”/ “no” |
| Discussions on social media (U) | “Did you participate in online discussions about the conflict in Ukraine last month?” | “yes”/ “no” |
| Online bubbles (C) | “How often did you spent time in online environments with people who strongly agreed with you on the issue of COVID-19 during the height of the pandemic?” | 1=”never”, 7=”more than 10 times a day” |
| Online bubbles (U) | “How often did you spent time in online environments with people who strongly agreed with you on the issue of the conflict in Ukraine last month?” | 1=”never”, 7=”more than 10 times a day” |
| Search for news (C) | “How often did you search for news about COVID-19 during the height of the pandemic?” | 1=”never”, 7=”more than 10 times a day” |
| Search for news (U) | “How often did you search for news about the conflict in Ukraine last month?” | 1=”never”, 7=”more than 10 times a day” |
| Sharing news (U) | “How often did you share news about COVID-19 during the height of the pandemic?” | 1=”never”, 7=”more than 10 times a day” |
| Sharing news (U) | “How often did you share news about the conflict in Ukraine last month?” | 1=”never”, 7=”more than 10 times a day” |
| Interest in news (C) | “How much were you interested in the issue of COVID-19 in the media during the height of the pandemic?” | 1=”I was not interested at all”, 5=”I was very interested” |
| Interest in news (U) | “How much were you interested in the issue of the conflict in Ukraine in the media last month?” | 1=”I was not interested at all”, 5=”I was very interested” |
| Frustration with the media | “To what extent do you agree or disagree with the following statement?”  “Public and mainstream media news usually frustrates or upsets me.” | 1=”strongly disagree”, 6=”I strongly agree” |
| **Political trust** |  |  |
| Trust in CZ-C | “How much do you trust...  1. The official statements of Ministry of Health regarding vaccination?  2. Czech public media to provide truthful information about COVID-19? | 1=”absolutely not”, 5=”absolutely yes” |
| Trust in CZ-U | “How much do you trust...  1. Czech government to take the right stance on the conflict in Ukraine?  2. Czech public media to provide truthful information about the conflict in Ukraine?" | 1=”absolutely not”, 5=”absolutely yes” |
|  | How much do you believe the states and international organizations listed below intentionally disseminate misleading information through social media? | 1=”never”, 5=”very often” |
| Distrust in Russia | “Russia” |  |
| Distrust in U.S. | “United States of America” |  |
| Distrust in E.U. | “European Union” |  |
| Distrust in China | “China” |  |
| Distrust in NATO | “NATO” |  |
| **Rigid beliefs** | “How often do you feel that your stance on societal issues (e.g., politics, war, pandemics, etc.) is so correct that no additional information could change your opinion?” | 1=”never”, 5=”very often” |
