# Supplementary material for: Beliefs in Misinformation About COVID-19 and the Russian Invasion of Ukraine Are Linked: Evidence From a Nationally Representative Survey Study
Source: JMIR Infodemiology. 2025 Mar 10;5:e62913. doi: 10.2196/62913 (PMC11956375; doi:10.2196/62913)
Supplement: Multimedia Appendix 2 [file infodemiology_v5i1e62913_app2.docx]

|  | | | | | | | | | |
| --- | --- | --- | --- | --- | --- | --- | --- | --- | --- |
|  | | Factor | | | | |  | | |
|  | | 1 | | | 2 | | Uniqueness | | |
| *BM-C.1 Western pharmacological vaccine companies* |  | | 0.207 |  | **0.625** |  | | 0.406 |  |
| *BM-C.2 Vaccines are dangerous for the vaccinated* |  | | 0.022 |  | **0.717** |  | | 0.467 |  |
| *BM-C.3 The discrimination against Russian vaccines* |  | | **0.559** |  | 0.150 |  | | 0.561 |  |
| *BM-C.4 The coronavirus was developed artificially* |  | | 0.161 |  | **0.524** |  | | 0.595 |  |
| *BM-C.5 The epidemic is fake* |  | | 0.025 |  | **0.798** |  | | 0.338 |  |
| *BM-C.6 Epidemic measures was ineffective* |  | | -0.132 |  | **0.806** |  | | 0.464 |  |
| *BM-U.1 Demilitarisation, de-Nazification of Ukraine* |  | | **0.787** |  | -0.026 |  | | 0.405 |  |
| *BM-U.2 The civilian casualties on the Ukrainian* |  | | **0.877** |  | -0.027 |  | | 0.260 |  |
| *BM-U.3 Ukraine is developing banned biological* |  | | **0.829** |  | 0.027 |  | | 0.285 |  |
| *BM-U.4 NATO and Western countries are exploiting* |  | | **0.793** |  | 0.023 |  | | 0.349 |  |
|  | | | | | | | | | |
|  | | | | | | | | | |
