# Supplementary material for: Beliefs in Misinformation About COVID-19 and the Russian Invasion of Ukraine Are Linked: Evidence From a Nationally Representative Survey Study
Source: JMIR Infodemiology. 2025 Mar 10;5:e62913. doi: 10.2196/62913 (PMC11956375; doi:10.2196/62913)
Supplement: Multimedia Appendix 3 [file infodemiology_v5i1e62913_app3.docx]

| Variable | SD | SE | Min | Max | M | Md |
| --- | --- | --- | --- | --- | --- | --- |
| Age | 15.56 | 0.39 | 20 | 91 | 55.04 | 57 |
| Education | 1.24 | 0.31 | 1 | 4 | 2.71 | 3 |
| Income | 1.15 | 0.03 | 1 | 4 | 2.89 | 3 |
| Gender | 0.50 | 0.01 | 1 | 2 | 1.52 | 2 |
| **BM-C**  1.  2.  3.  4.  5. | 5.78  1.15  1.16  1.26  1.23  1.17 | 0.14  0.03  0.03  0.03  0.03  0.03 | 5  1  1  1  1  1 | 25  5  5  5  5  5 | 12.11  2.60  2.41  3.12  2.33  2.85 | 13  3  2  3  2  3 |
| **BM-U**  1.  2.  3.  4. | 4.26  1.21  1.23  1.14  1.33 | 0.11  0.03  0.03  0.03  0.03 | 4  1  1  1  1 | 20  5  5  5  5 | 9.89  2.11  2.45  2.41  2.91 | 10  2  3  3  3 |
| COVID-19 vaccination | 1.17 | 0.03 | 0 | 3 | 2.18 | 3 |
| Emails | 1.36 | 0.03 | 1 | 6 | 2.21 | 2 |
| YouTube | 1.50 | 0.04 | 1 | 6 | 2.43 | 2 |
| Anti-system websites | 1.18 | 0.03 | 1 | 6 | 1.71 | 1 |
| Public media | 1.62 | 0.04 | 1 | 6 | 3.51 | 4 |
| Mainstream news websites | 1.66 | 0.04 | 1 | 6 | 3.48 | 4 |
| Exposure to social media | 107.30 | 2.66 | 0 | 1440 | 101.82 | 80 |
| Social media info. source | 1.24 | 0.03 | 1 | 5 | 2.90 | 3 |
| Discussions under web news | 1.07 | 0.03 | 1 | 5 | 2.78 | 3 |
| Discussions social media (C) | 0.37 | 0.01 | 1 | 2 | 1.16 | 1 |
| Discussions social media (U) | 0.28 | 0.07 | 1 | 2 | 1.09 | 1 |
| Online bubbles (C) | 1.22 | 0.03 | 0 | 7 | 0.48 | 0 |
| Online bubbles (U) | 0.10 | 0.02 | 0 | 7 | 0.27 | 0 |
| Search for news (C) | 1.32 | 0.03 | 1 | 7 | 3.03 | 3 |
| Search for news (U) | 1.45 | 0.04 | 1 | 7 | 2.99 | 3 |
| Sharing news (C) | 1.10 | 0.03 | 1 | 7 | 1.64 | 1 |
| Sharing news (U) | 1.02 | 0.03 | 1 | 7 | 1.52 | 1 |
| Interest in news (C) | 0.89 | 0.02 | 1 | 4 | 2.31 | 2 |
| Interest in news (U) | 0.92 | 0.02 | 1 | 4 | 2.90 | 3 |
| Frustration with media | 1.26 | 0.03 | 1 | 6 | 3.96 | 4 |
| **Trust in CZ (C)**  1. Czech Ministry of Health  2. Public media | 2.12  1.10  1.14 | 0.05  0.03  0.03 | 2  1  1 | 10  5  5 | 5.60  2.90  2.70 | 6  3  3 |
| **Trust in CZ (U)**  1. Czech government  2. Public media | 2.46  1.31  1.25 | 0.06  0.03  0.03 | 2  1  1 | 10  5  5 | 5.38  2.68  2.69 | 6  3  3 |
| Distrust in Russia | 1.16 | 0.03 | 1 | 5 | 3.98 | 4 |
| Distrust in U.S. | 1.10 | 0.03 | 1 | 5 | 3.30 | 3 |
| Distrust in EU | 1.15 | 0.03 | 1 | 5 | 3.11 | 3 |
| Distrust in China | 1.16 | 0.03 | 1 | 5 | 3.68 | 4 |
| Distrust in NATO | 1.18 | 0.03 | 1 | 5 | 3.02 | 3 |
| Rigid beliefs | 1.05 | 0.03 | 1 | 5 | 2.94 | 3 |
